# The Relationship between Cognitive Impairment and Violent Behavior in People Living with Schizophrenia Spectrum Disorders: A Critical Review and Treatment Considerations

**DOI:** 10.3390/medicina60081261

**Published:** 2024-08-03

**Authors:** Gabriele Nibbio, Lorenzo Bertoni, Irene Calzavara-Pinton, Nicola Necchini, Stefano Paolini, Antonio Baglioni, Daniela Zardini, Laura Poddighe, Viola Bulgari, Jacopo Lisoni, Giacomo Deste, Stefano Barlati, Antonio Vita

**Affiliations:** 1 Department of Clinical and Experimental Sciences, University of Brescia, 25123 Brescia, Italy; gabriele.nibbio@gmail.com (G.N.); lorenzo.bertoni93@gmail.com (L.B.); nnecchini@gmail.com (N.N.); s.paolini@unibs.it (S.P.); a.baglioni@unibs.it (A.B.); dani.zardi@gmail.com (D.Z.); viola.bulgari@unibs.it (V.B.); giacomodeste@mac.com (G.D.); antonio.vita@unibs.it (A.V.); 2 Department of Mental Health and Addiction Services, ASST Spedali Civili of Brescia, 25123 Brescia, Italy; irene.calzavarapinton@gmail.com (I.C.-P.); laura.poddighe@outlook.com (L.P.); jacopo.lisoni@gmail.com (J.L.); 3 Department of Mental Health, ASST Valcamonica, 25123 Brescia, Italy

**Keywords:** aggressive behavior, cognition, cognitive remediation, physical exercise, schizophrenia spectrum disorders, social cognition, violence

## Abstract

Cognitive impairment is a core feature of schizophrenia spectrum disorders (SSD). Violent and aggressive behavior represents a complex issue in psychiatry, and people with SSD have been shown to be at risk of being both victims and perpetrators of violence. In this review, the complex relationship between cognitive impairment and violent behavior is explored, also considering the usefulness of treating cognitive impairment to improve violence-related outcomes. Several studies report that cognitive impairment is linked to violent behavior, but significant differences between domains and conflicting results are also present, leaving the identification of specific cognitive profiles predicting violent behavior in SSD as an important aim for future research. Evidence regarding the effectiveness of treating cognitive impairment to improve violent behavior, while heterogeneous, provides more consistent results: cognition-targeting interventions appear to provide significant benefits also in the prevention of aggression in people living with SSD, and preliminary evidence shows cognition-focused interventions targeting violent behavior improve both cognition- and violence-related outcomes. Implementing these interventions in clinical practice could be of great usefulness, particularly in forensic contexts. Physical exercise, which improves cognitive performance and psychosocial functioning in SSD, appears to reduce violent behavior in healthy individuals, but requires further studies in clinical samples.

## 1. Introduction

Compared to the general population, people living with mental disorders are more frequently involved in episodes of violence; this issue appears to be even more evident in people living with schizophrenia spectrum disorders (SSD), which are more likely to be the victims of violent episodes and, concurrently, the perpetrators of violent behaviors, both when compared to healthy controls, but also when compared to people living with other mental disorders [1,2,3,4,5,6].

The relationship between SSD and violent behavior is complex and mediated by multiple factors, such as demographic variables, clinical variables, and neurobiological variables. More specifically, various studies have highlighted anatomical and physiological differences between violent and non-violent individuals living with SSD: brain alterations involving hippocampus, amygdala, and frontal cortex, assessed through fMRI, have been hypothesized to correlate with aggressive and violent behaviors [7,8]. These data are of considerable interest in a research perspective, but are still far from being impactful in the clinical context and to be usefully implemented in the day-to-day practice of mental health services and in forensic settings. Moreover, although violent behavior in people living with SSD appears to be influenced by several variables, the ones more specifically associated to SSD are positive symptoms (in particular persecutory delusions and hallucinations), lack of insight, and non-adherence to psychopharmacological treatments [9,10]. Several other risk factors are shared with the general population: the most relevant ones are represented by previous episodes of violence [11], substance abuse, homelessness, being violently victimized, parental violence and male gender [9,12].

The association between cognitive dysfunctions and increased risk of violent behaviors in people living with SSD is an issue of growing interest both in a scientific and in a clinical perspective. In general, cognitive deficits are a core element of SSD: they include both neurocognitive and social cognition domains [13,14,15], they can be observed from young ages, even predating the clinical onset of the illness [16], and they have a strong negative impact on real-world psychosocial functioning [17,18]. Deficits in global cognitive performance have also been hypothesized to represent an important predictive factor for violent behaviors, but the available literature presents heterogeneous results, particularly as regards the effects of specific domains [9,19,20,21]. In particular, it appears that people living with SSD showing a history of violent behaviors, on average, score lower in a variety of cognitive domains, suggesting that impaired cognitive functions could favor the presence of violent behaviors [22,23]; however, different studies report conflicting results, and it is possible that the relationship between cognitive impairment and violent behavior may be more articulated and nuanced.

The aim of the present narrative and critical review was to investigate the complex relationship between violence and cognition in people living with SSD, exploring and commenting the results of the most recent and impactful studies, aiming also to better define the clinical implications of this relationship and to consider this issue in a treatment perspective. In fact, a deeper understanding of the mechanisms regulating these two factors could help clinicians to assess more accurately the risk of violent behaviors in people living with SSD and to implement interventions that could possibly help to prevent them both in a direct and in an indirect manner.

## 2. Materials and Methods

To identify studies exploring the relationship between cognition and violence in people living with SSD, investigating also the potential effects of treatments targeting cognitive performance, searches were conducted on electronic databases PubMed, Scopus, and Google Scholar from 3 May 2024, since inception.

Key terms for the searches included “violence” or “violent behavior” or “aggression” or “aggressive behavior” and “cognition” or “cognitive performance” or ”cognitive remediation” or “physical exercise” and “schizophrenia”. While violent behavior and aggressive behavior are not equivalent, both were considered of interest.

Narrative reviews form the basis of medical literature synthesis. Unlike scoping reviews, they can consider several different questions at a time, and unlike systematic reviews they can include the opinions, perspectives, and speculations of experts beside and beyond the evidence reported in primary research. In this context, they can provide a critical assessment of the available evidence and offer valuable insight for future research [24,25,26,27].

Both original studies and reviews were considered of interest for the purpose of the present work, as reviews usually also include assessments, perspectives, and comments by the authors and therefore a narrative and critical perspective could provide valuable information beyond the sum of the included studies. Literature emerging from the searches was assessed by at least two investigators for inclusion in the review. References of interest emerging from the citations of included works were also taken into account for inclusion.

As the selection of the included literature did not follow a systematic procedure, given the structure and the aims of the present review, it should be noted that the studies present in the review to do not represent the totality of the works investigating the explored topic.

## 3. Results

### 3.1. Violent Behavior and Cognitive Performance

Cognitive impairments commonly observed in SSD have been associated with an increased risk of violent behaviors: this paragraph will report the most recent and significant literature studies that analyze the relationship between cognitive functioning and violent behaviors in people living with SSD.

In 2014, Reinharth and colleagues conducted a meta-analysis to investigate the correlation between cognitive impairment and violence in patients living with SSD. They identified 29 studies, with a total sample of 4764 subjects which presented heterogeneous characteristics and were evaluated with a variety of neuropsychological tests [21]. Most of the selected studies included male subjects, with the assumption that female patients would be less prone to show violent behaviors, although some previous studies reported that female patients were more likely to enact aggression compared to male patients [28]. The meta-analysis showed that cognitive impairment was an important risk factor for violent behavior; more in detail, an impaired global cognition (as measured by the Wechsler Adult Intelligence Scale—WAIS and the cognitive subscale of the Positive and Negative Syndrome Scale—PANSS) and a poor insight were the factors more strongly correlated to a higher likelihood of violent acts. However, the association between worse cognitive performance and risk of violence was not observed for all cognitive domains: for instance, a better performance in motor functioning was correlated with a higher likelihood of aggression. Visuospatial reasoning performance was inversely associated with violent behaviors, but this result did not reach the significance threshold, while memory and attention did not seem to show any correlation with aggression. Finally, the included studies showed that worse cognitive functioning during hospitalization was correlated with a higher likelihood of violent behavior after discharge and a higher likelihood of aggressive behavior before hospitalization. In general, the results of the meta-analysis showed that among people living with SSD a greater neurocognitive impairment is correlated with a higher risk of violent acts and, in particular, people with a global cognition score that is more than one standard deviation below the mean, especially if associated to poor insight, are at higher risk of enacting violent behaviors, both in hospital and in community settings [21].

In 2015, a study from O’Reilly et al. investigated the relationship between neurocognition, social cognition, and violence in 89 forensic patients diagnosed with SSD [29]. Participants with violent behavior showed significantly lower scores in the social cognitive reasoning task (as measured with the Mayer–Salovey–Caruso Emotional Intelligence Test—MSCEIT item of the MATRICS Consensus Cognitive Battery—MCCB) with respect to other participants. Additionally, the authors found that social cognition appears to have a direct effect on violence independently from other variables, whereas the relationship between neurocognition and violence appears to be mediated by social cognition (as measured by the MSCEIT item of the MCCB), symptom severity (as measured by the total score of the PANSS), social functioning (as measured by the Social and Occupational Functioning Assessment Scale score) and violence proneness (as measured by the total score of the Historical Clinical Risk Management-20—HCR-20) [29].

O’Reilly et al. conducted another study published in 2020, including a sample of 55 participants diagnosed with SSD, again recruited in a forensic setting [30]. The assessment included a wide variety of measures: the Schedule for the Assessment of Psychotic Symptoms, the HCR-20, the MCCB and an expert assessment of moral cognitions based on Haidt’s moral foundations theory. While the study was more heavily focused on moral cognition aspects and no dedicated analysis explored the relationship between cognitive scores and violent behavior, instead using MCCB scores as a controlling factor in other analyses, this forensic sample showed a mean t-score of 26, suggesting the presence of significant cognitive impairment [30].

Another important study was published in 2020 by Lamsma et al.: this study included a large sample of 891 participants diagnosed with SSD and recruited in the Netherlands as part of the Genetic Risk and Outcome of Psychosis research project [31]. Participants were assessed with a wide battery of neuropsychological tests, including the Continuous Performance Test, the Response Shifting Task, the WAIS Third Edition Block Design Subtest, and the Neuropsychological Assessment Battery (NAB) Mazes Test to assess neurocognitive performance and the Degraded Facial Affect Recognition Task and the Hinting Task to assess social cognitive performance. Violent behavior was assessed using the Life Chart Schedule, with a lifetime reference period, and was observed in 21% (*n* = 183) of the sample, while 79% (*n* = 615) of the sample showed no history of aggression. Participants with violent behavior showed a significantly worse performance in the WAIS Block Design Subtest, in the NAB Mazes Test, and the Hinting Task, albeit in all cases with small effect sizes. Worse performances in participants with violent behavior were also observed in most of the other neuropsychological tests, but these differences did not reach the threshold for statistical significance [31].

In a 2021 study by Iozzino et al., neurocognition and social cognition were assessed in participants living with SSD with (221 subjects) and without (177 subjects) a history of violence [32]. In this large multinational European study patients were recruited from both forensic and general psychiatric settings and for each participant several variables were evaluated: symptom severity using the PANSS scale, psychosocial functioning using the World Health Organization Disability Assessment Schedule 2.0, neurocognition using the Brief Assessment of Cognition in Schizophrenia (BACS) Test and social cognition using an Emotion Recognition Task and a Story-based Empathy Task. The results of the study report that the strongest discriminators between participants with a history of violence and without a history of violence were education and processing speed (as measured with the BACS-Symbol Coding Task), followed by emotion recognition and, more specifically, increased accuracy for anger recognition seemed to be the most accurate distinctive factor for the forensic group [32].

More recently, Okasha et al., conducted and published a study that included a sample of healthy controls and a sample of 50 individuals diagnosed with schizophrenia, both recruited in Egypt [33]. Participants diagnosed with schizophrenia were assessed with the HCR-20 scale, the PANSS, the WAIS in its Egyptian translation, the Trail Making Test (TMT) Part A and B, the Wisconsin Card Sorting Test (WCST), and the Wechsler Memory Scale (WMS). According to HCR-20 scores, the authors reported a significant risk of violent behavior in 58% (*n* = 29) of participants with schizophrenia: these participants showed a significantly worse performance in the WCST total correct answers, WCST total errors, WCST conceptual level responses, WCST percent of conceptual level responses, WCST categories completed, and WCST trials to complete first category, but no substantial difference was observed in other tests. The total PANSS score and the history of substance use emerged as the only individual predictors of violence risk in regression analyses; these results, however, have to be considered also in light of the very limited sample size included in the study [33].

A study by Barlati et al. compared clinical, cognitive, and psychosocial functioning parameters in 50 subjects living with SSD convicted for violent crimes and 50 subjects living with SSD with a negative history for violent crimes [20]. Offenders showed greater deficits in the working memory domain (measured by the TMT Part B Test) and in processing speed domain (measured by the STROOP test time and the BACS Token Motor Test), compared to non-offenders. In contrast, violent offenders showed better attentional performance (assessed by STROOP test errors). Individual predictors of violent behavior were investigated using logistic regression analyses: better attentive performance as measured by the Stroop test errors and worse performance in speed of processing as measured by the Stroop test time and BACS Token Motor Test emerged as cognitive predictors, alongside the number of school failures, the severity of excitatory symptoms, the HCR-20 Risk Management subscale score and the Psychopathy Checklist-Revised (PCL-R) “Callous” Factor score which emerged as predictors in models dedicated to clinical, sociodemographic, and aggression-related variables. Post hoc analyses also showed that better scores in the attention domain were associated with a higher risk of violence relapse (as measured by the HCR-20 scale), a higher level of psychopathy (in both factors of the PCL-R scale), and a lower level of impulsivity (assessed by the Barratt Impulsiveness Scale—BIS-11 subscale). Furthermore, it was found that greater deficits in several cognitive profiles were associated with more evident antisocial behaviors (PCL-R ‘unstable’ factor) [20].

In another recent study, conducted in 2023 by Vaskinn et al., social cognition parameters were analyzed using the Emotion in Biological Motion (EmoBio) and the Movie for the Assessment of Social Cognition (MASC) tests [23]. Participants were stratified in four groups for a total of 167 male participants divided as follows: 22 violent offenders (homicide, attempted homicide, severe violence, and sexual offences) (V group), 27 subjects living with SSD and with a history of interpersonal violence (SSD-V group), 42 individuals living with schizophrenia with a negative personal history for aggression (SSD-NV group), and 76 healthy controls included in a previous study (HC). The results of the study show that subjects with a history of violent behaviors (V and SSD-V) perform worse in social cognition parameters compared to non-violent subjects (SSD-NV and HC). However, it is not yet clear whether there is a difference in social cognition parameters between people with a history of violent behavior with or without a diagnosis of SSD. In particular, it emerged that the SSD-V group was found to have a greater impairment than the SSD-NV group with regard to total score and anger in the EmoBio and had worse total scores than the HC group in the MASC, without however showing significant differences between the V and the SSD-V groups. In the SSD-V group, a greater impairment was observed in the total score, the neutrality score, and the fear score, whereas the impairment was slightly lower in the V group. No significant differences were shown in EmoBio scores when comparing the SSD-NV group and the HC group. Vaskinn and coworkers also observed that social cognition deficits appear to characterize subjects with a history of violence regardless of whether they have a diagnosis of SSD; however, the degree of the impairment seems to be influenced by the presence of an SSD diagnosis. Moreover, subjects living with SSD and a positive history of violent behavior had a more extensive impairment regarding social cognition parameters compared to people living with SSD and without history of violence. Interestingly, the only predictor of violence identified was the Theory of Mind domain and having a diagnosis of SSD did not correlate with being in the violent group or not [23].

Another recent study conducted in China by Yi et al. included a total of 337 inpatients diagnosed with schizophrenia and assessed using the Repeatable Battery for the Assessment of Neuropsychological Status (RBANS) and the PANSS [34]. Episodes of violent behavior during the hospitalization were investigated through direct questioning of participants, and the sample was divided into participants with (*n* = 35) and without (*n* = 302) episodes of violence. Participants with episodes of violence showed a worse global cognitive performance as measured by the total RBANS score and a worse performance in the language component of the RBANS, as well as a more severe cognitive symptoms as measured by the PANSS cognitive factor scores; worse symptoms were also observed in this group as measured by the total PANSS score and by scores of the Positive and Excited factors. Performance at the language component of the RBANS also emerged as an individual predictor of violent behavior in a binary logistic regression analysis, alongside other recognized sociodemographic and clinical factors such as male gender, illness duration, positive symptoms severity, and tobacco smoking. Despite the limitations of the study, consisting mainly in its cross-sectional structure and in the restricted and subjective assessment of violent and aggressive behavior, these results further confirm the relationship between worse cognitive performance and increased risk of violence in people living with SSD [34].

Finally, a recent systematic review and meta-analysis conducted by Griffith et al. and published in 2024 explored the relationship between executive functioning and violent behavior measured as involvement with justice related to aggression [35]. While this systematic review and meta-analysis did not exclusively include participants diagnosed with SSD and the included studies presented a high level of heterogeneity, a significant relationship with a medium-sized effect (d = 0.55) linking executive dysfunction and violent behavior was observed. These results provide additional evidence confirming the relationship between cognitive deficits and violence, which were reported and investigated in people with SSD in the aforementioned studies.

### 3.2. Treating Cognitive Performance to Improve Violent Behavior

The observation of a strong associations between cognitive impairment and violence has led the scientific community to develop the hypothesis that interventions targeting cognitive performance could also be a useful tool in the in prevention and treatment of violent behavior [36,37]. Among possible treatments, evidence-based psychosocial interventions such as cognitive remediation (CR) [27,38,39,40] and physical exercise (PE) [41,42,43,44] have been shown to have consistent evidence of effectiveness on cognitive performance and are recommended for this outcome in the most recent European Psychiatric Association guidance [17]; in this paragraph we summarize the most relevant studies analyzing the efficacy of these interventions on violence in people living with SSD.

In a 2017 systematic review, Darmedru et al. evaluated the effectiveness of CR and social cognitive training in decreasing violent acts in individuals living with schizophrenia; a total of 11 studies were included and it was concluded that both CR and social cognitive training were effective in decreasing violent behavior in patients living with SSD [35]. Among the included studies, two of them demonstrated the effectiveness of short [45] and comprehensive [46] CR in decreasing violent episodes in outpatients and inpatients diagnosed with SSD, while the improvement emerged at all stages of the disorder and was correlated to an improvement in neurocognition. Moreover, two studies showed that SCIT (Social Cognition and Interaction Training), a cognitive-behavioral group therapy that aims to improve subjects’ social cognition functions, was effective in reducing aggression in subjects diagnosed with SSD [47,48]. Also, five other studies showed a reduction in aggression in subjects living with SSD who participated in the R&R-2 MHP intervention (revised version of Reasoning and Rehabilitation program), which was effective in decreasing impulsivity and verbal aggression [49,50], and physical aggression [36]. Finally, two studies analyzed the benefits of Metacognitive Training (MCT), which was observed to be effective in decreasing symptoms related to violent behaviors such as tension, hostility, arousal, and aggressive behaviors in general [36]. In summary, this systematic review found that cognitive remediation and social cognitive training were shown to be effective in decreasing violent acts in patients with SSD at various stages of the disorder; in fact, the goal of these interventions is to decrease aggression by providing improvements in executive functions, verbal memory, and social cognition and in some clinical parameters such as impulsivity, hostility, and arousal [36].

Jones and Harvey also considered the effectiveness of CR and social cognitive training on violent and aggressive behavior in people living with SSD in a non-systematic review published in 2020 [37]. While this review was conducted using a non-systematic approach, it remarked that the results of 15 studies as effects on determinants of violence, such as substance use and certain measures of psychosocial functioning, were also taken into account. In this review, the mechanisms through which CR and cognition-oriented interventions could have a positive impact on violent behavior are discussed in detail, leading the authors to conclude that CR and social cognitive training may represent the interventions with the greatest potential to reduce violent behavior and aggression in people living with SSD.

A study published in 2022 and conducted by Hsu and Oyang investigated whether an intervention specifically targeting violent behavior in people with schizophrenia also had a positive effect on other outcomes such as alexithymia and cognition [51]. In this study, a sample of 60 participants was randomized with a 1:1 proportion to receive a psychosocial intervention focused on the prevention of violent behavior that also included several elements of neurocognitive and social cognition training or to receive treatment as usual. Significant positive effects were observed at post-intervention and a follow-up observation in several aggression-related parameters measured with the Modified Overt Aggression Scale and the Buss–Perry Aggression Questionnaire, including verbal aggression, physical aggression, resentment, and hostility, at the Cognitive Failure Questionnaire, in several items of the Rationale-Experiential Inventory and in emotional regulation parameters such as the suppression subdomain. Other items of the Rationale-Experiential Inventory and the reappraisal subdomain of emotional regulation showed significant between-group differences that emerged in the follow-up assessment. While the assessment of cognitive performance was not conducted using highly recommended cognitive assessment batteries that allow the observed results to be easily reproduced and compared [18], these results further confirm the link connecting cognitive performance and violent behavior in people living with SSD and highlight the effectiveness of treating both aspects with cognition-oriented approaches [51].

More recently, a feasibility study to investigate the efficacy of immersive videos to treat social cognitive impairment in people with schizophrenia recruited in a forensic setting was developed [52]. While this study did not include a control group, it led to the design and the validation of the videos included in the intervention and allowed attestation, in a preliminary sample of seven participants, that the intervention has a good feasibility and tolerability profile.

PE represents another important evidence-based psychosocial intervention that can lead to significant improvements in both positive and negative symptoms, psychosocial functioning, and cognitive performance in people living with SSD [42,43,53,54,55]. PE interventions can be also easily combined with CR interventions in integrated treatment programs that provide superior benefits compared to each of its components alone [56,57,58].

Moreover, PE interventions appear to be consistently effective in reducing violent behaviors in non-clinical samples [59,60].

Fazel et al. conducted an umbrella review on the effectiveness of violence-prevention interventions published in 2023 [59]. A total of 16 meta-analyses exploring different types of interventions were included in the meta-review; while most interventions showed some measure of effectiveness in reducing violent behaviors, PE-based interventions appeared to provide the largest benefits.

Two meta-analyses, one published in 2016 including any type of PE [61] and one published in 2017 and focused on martial arts sports [62], investigated the effects of these interventions on violence-related outcomes in children and adolescents, observing consistent positive effects in both instances.

More recently, another meta-analysis, conducted by Ouyang and Liu and published in 2023, investigated the effectiveness of PE on aggressive behaviors in children and adolescents: in a total of 15 studies, significant medium-sized positive effects were observed on total aggression scores as measured by external observers and large-sized positive effects were observed on hostility in participants that showed tendency to aggression [60].

However, to the best of our knowledge, there are no studies, thus far, specifically examining the efficacy of PE as a possible prevention measure or treatment for aggressive or violent behavior in SSD.

A recent systematic review assessed the effects of PE interventions in people living with severe mental illnesses, including SSD, in forensic settings [63]: positive effects were consistently reported in metabolic and anthropometric variables such as weight and waist circumference, and PE emerged as superior to control (but inferior to token-behavior therapy) in the negative symptoms dimension in one study [64]. However, aggression or violent behavior were not taken into account as outcomes in any of the included studies.

In light of the fact that PE seems to be effective in non-clinical populations in preventing violent and aggressive behavior [59,60] and of the observations regarding the contribution of cognitive deficits to violent behavior, as well as taking into consideration that PE seems to be effective in improving cognitive performance, a role of PE in the prevention and treatment of aggression and violent behavior in people living with SSD appears as a promising perspective. Dedicated studies should be devised to assess specifically the effectiveness of PE intervention, or of integrated rehabilitation programs that include PE, including also violent behavior and aggression as a principal study outcome.

## 4. Discussion

In the present review, we analyzed the most recent and significant evidence concerning the relationship between cognition and violence in patients living with SSD.

**Figure 1:**
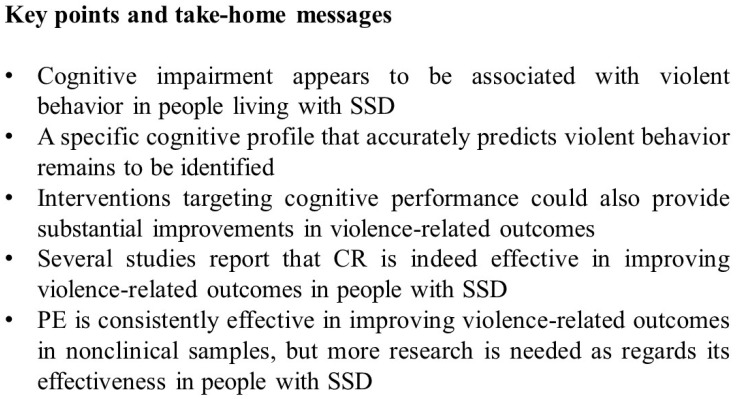
**Key point and take-home messages of the review.** CR: cognitive remediation; PE: physical exercise; SSD: schizophrenia spectrum disorders.

Key points and take-home messages of the present work are presented in Figure 1.

According to the 2014 metanalysis by Reinharth and coworkers, cognitive impairment explains roughly 2% of the variance in observed violence, and people living with SSD having an overall performance at least 1 SD lower than the mean are at higher risk of perpetuating violent behaviors [21].

Focusing on specific cognitive domains, it appears that executive functions have a central role in the process linking violence and cognition: executive functions appear to be involved in the modulation of aggression [65] and also in the control of the inhibitory response [66]. Moreover, deficits in the executive functions have been shown to be correlated to increased aggression [67,68,69,70].

Additionally, deficits in the memory functions appear to be associated to violent behavior: several studies show that patients living with SSD with a worse performance in verbal encoding speed and in the amount of encoded information seem to have a relatively lower ability to store new information and to retrieve this information in the future; this difficulty could be related to a possible exacerbation of delusions when the patient feels contradicted or questioned about such notions [67,68]. A correlation with violent behavior was shown in subjects with deficits in attention [67,69], processing speed, and motor speed [65], while social cognitive deficits also appear to play a relevant role [71,72,73].

Moreover, lack of insight seems to be one of the main predictors of violence, and not only lack of insight related to illness awareness—thus being able to recognize the falseness of delusions and hallucinations—but also lack of insight related to the need and benefits of psychopharmacological treatment, resulting in poor medication adherence, and consequently having a negative impact on psychosocial functioning [74,75,76,77].

Therefore, in light of these results, it appears that impaired cognitive functioning may be correlated to an increased predisposition to violence and aggression. However, not all the studies analyzed present homogeneous results;, for instance, in the study by Barlati et al., SSD offenders presented lower scores in the domains of processing speed and verbal memory than non-offenders, although the former also presented better attentional scores than the latter. This finding has been hypothesized to have different reasons, that could be explained by the presence of specific cognitive patterns in offenders, due to the cognitive heterogeneity between the included subjects or by the fact that forensic patients may have approached the neuropsychological test with greater participation intent than controls [20,21,75,78,79].

Moreover, in the study led by Candini et al. [80] and the one led by Bulgari et al. [81], the results state that patients living with SSD with violent behaviors had better cognitive performances than control subjects, both in terms of executive functioning and motor tasks. The authors speculate that a possible explanation for their results could be that patients with a positive history of violence had a lower severity of negative symptoms [82].

On the other hand, other studies found no statistically significant differences regarding overall cognitive performance between individuals living with SSD with or without history of violent behavior [83,84].

Comparing the results from different studies showed that they are heterogeneous regarding cognitive functioning and its relation to violent behaviors in people living with SSD, but the heterogeneity of such results highlights the need to delineate better the cognitive characteristics of people with SSD which are more prone to show violent behaviors, with the aim of identifying tailored treatment and prevention strategies for violence [13,20,84,85]. Recent evidence also suggests that, while the total number of episodes of violence perpetrated by people living with SSD is decreasing, probably as a result of a more consistent implementation of better therapeutic strategies and interventions, the rate between episodes of violences perpetrated by people living with SSD and those perpetrated by the general population is increasing, suggesting that specific factors linked to the condition remain to be adequately addressed [6].

In this context, a few literature studies have shown a significant efficacy of some anti-psychotic drugs such as clozapine in treatment of violence [86]. Olanzapine has also been seen to improve cognitive performance and reduce aggression in patients living with SSD [86]. The aforementioned antipsychotics have been hypothesized to be effective on aggressive behaviors due to a more selective action on serotoninergic and cholinergic neurotransmission in limbic and prefrontal areas [87].

CR, on the other hand, has been proven to be the most effective treatment for cognitive deficits in people living with SSD, and it is a recommended treatment in the most recent European Psychiatric Association guidelines [17]. More specifically, CR was demonstrated to reduce both verbal and physical aggression in patients living with SSD with or without a history of violent behaviors, as well as showing significant effectiveness on different cognitive domains [35,46,49,88]. The mechanism by which CR has been shown to reduce violent behavior does not seem to be exclusively related to improvement of cognitive performances, but also to a reduction in hostility, impulsivity, and arousal parameters [32]. CR and social cognitive training could also help participants in the development of novel cognitive strategies that could offer, in critical contexts, alternatives to the use of impulsive, aggressive and violent reactions [36]. The fact that the most common form of aggression perpetrated by people living with SSD appears to be the reactive-impulsive type, which appears to be related to an emotion-driven impulsivity mechanism or to an alteration in emotion generation in limbic areas [89], could explain the efficacy of CR in reducing aggressive and violent behaviors, prompting clinicians to implement this type of treatment in routine clinical practice particularly in the forensic setting [17,36,37]. CR and social cognition training also help in development of novel skills and have substantial positive effects in real-world psychosocial functioning [38,39,40]; this could also have indirect positive repercussions on the likelihood of recurring to violent reactions for participants [37].

Moreover, PE has also been linked to improvements in cognitive abilities and more specifically in the domains of psychomotor speed, social cognition, attention, and working memory [41,89,90]. This appears to be related to an increase in neurogenesis and neuronal plasticity through a mechanism of increased release of brain-derived neurotrophic factors [91,92]. Different psychosocial interventions have been shown to be effective in preventing violent behavior in non-clinical samples, with PE-based programs representing the intervention providing the largest benefits [57]. Considering that PE is a well-recognized evidence-based intervention for SSD [43,93], that has also been shown to provide reliably improvements in personal and social functioning [40], it could represent a very valuable treatment in forensic settings and for people living with SSD with a history of violent offence more in general. Further studies are needed to demonstrate a correlation between improvement in cognitive abilities through PE and reducing violent behavior, specifically in people with SSD.

Although the studies discussed in the present review show a significant link between cognition and violent behavior, some limitations should be stated. First of all, although a correlation between violent behavior and cognition in people living with SSD was highlighted in several studies, the criteria used to define aggression were very heterogeneous, as some works considered as violent acts only physical aggressions toward others, whereas other works also included in the definition of violent acts verbal aggressions, aggression towards inanimate objects, and self-aggressiveness. Considering this heterogeneity, the use of a more specific and rigorous methods in defining violent behavior is needed to provide a better standardization of results in future studies [21,36]. Second, the assessment of cognitive functions also showed a very important level of heterogeneity, both considering the variety of neuropsychological tests and the evaluation and analysis of different cognitive domains [21,36]. The heterogeneity in the evaluation of cognition could have led to an increased variability of the outcomes, with the consequent risk of substantially increasing the difficulty of establishing a precise correlation with violent behavior. In this regard, future studies should be designed considering the need to standardize and compare the observed findings, and should consider in a more consistent manner the use of validated and recommended assessment batteries to evaluate cognitive outcomes [18]. A lack of homogeneity was also observed in the study populations; for instance, several studies included subjects with comorbid substance use disorder and antisocial personality disorder. Moreover, some studies excluded subjects with comorbidities such as intellectual disabilities or traumatic brain injuries, whereas other studies did not specify exclusion criteria for these parameters. Some studies had small sample sizes and a limited setting, with consequent reduction in generalizability of the results [46,68]. In addition, the differences that emerged between the participants, both with regard to symptom severity and level of baseline cognitive performance, may have partially led to the observation of conflicting results [82].

Finally, a limitation of the present review is that, as it followed a narrative approach [24,25], it was not based on a systematic literature search and was not conducted using a systematic approach. Moreover, as it tackled different inter-related topics, it could not be construed as a scoping review. As such, it did not adhere to PRISMA recommendations for scoping and systematic reviews [94,95].

## 5. Conclusions

In conclusion, this narrative and critical review highlighted the evidence linking cognitive functioning and aggressive behaviors in people living with SSD as well as the studies showing that evidence-based psychosocial interventions have a strong potential in the prevention of aggression.

The key points and take-home messages of the review are that cognitive impairment appears to be consistently associated with violent behavior in people living with SSD, but a specific cognitive profile that accurately predicts violent behavior remains to be identified.

As regards interventions targeting cognitive performance such as CR and PE, they could also provide substantial improvements in violence-related outcomes.

In fact, several studies report that CR appears to be effective in reducing both verbal and physical aggression in patients living with SSD. PE is consistently effective in improving violence-related outcomes in nonclinical samples, but more research is needed to assess its effectiveness in people living with SSD.

Future research efforts should be made to identify specific cognitive profiles linked to aggressive and violent behaviors by evaluating subjects with standardized and specific measures. The identification of specific cognitive profiles represents a game-changer both in preventing aggressive behavior and in identifying targeted therapeutic interventions and tailored rehabilitation interventions.

With this in mind, future research should continue to focus on identifying cognitive profiles linked to violent behaviors in people living with SSD through the development of qualitatively superior and more easily replicable studies, with the aim of consolidating the results obtained thus far and making them more viable for clinical practice. Future studies investigating the cognitive and violence-related effects of different evidence-based psychosocial interventions could also provide more valuable information regarding the development, the implementation, and the personalization of effective treatment and rehabilitation programs, both in routine clinical practice and in specific forensic settings.

## Data Availability

The data that support the findings of this study are available from the corresponding author upon reasonable request.
